# Exploring Healthcare Staff Perceptions and Satisfaction with the Physical Work Environment: A Qualitative Study

**DOI:** 10.3390/healthcare14050642

**Published:** 2026-03-04

**Authors:** Roshan S. Shetty, Giridhar B. Kamath, Sham Ranjan Shetty, Sriram KV, Akshatha Rao, Vibha Prabhu, Smitha Nayak

**Affiliations:** 1 Manipal School of Architecture and Planning, Manipal Academy of Higher Education, Manipal 576104, India; roshan.shetty@manipal.edu (R.S.S.); akshatha.rao@manipal.edu (A.R.); 2 Manipal Institute of Technology, Manipal Academy of Higher Education, Manipal 576104, India; giridhar.kamath@manipal.edu (G.B.K.); kv.sriram@manipal.edu (S.K.); vibha.prabhu@manipal.edu (V.P.); 3 T. A. Pai Management Institute, Manipal Academy of Higher Education, Manipal 576104, India; shamranjan.shetty@manipal.edu

**Keywords:** healthcare facilities, workplace environment, healthcare staff, greenspaces, safe workplace, well-being

## Abstract

Background: This study explores how healthcare staff perceptions of their physical work environment influence their satisfaction. Methods: A qualitative research design involving semi-structured interviews was adopted. The study sample comprised ten healthcare staff, including both clinical and nonclinical employees, working in a healthcare facility. The participants represented a range of professional roles and work areas, allowing for diverse perspectives on the physical environment. The data were analyzed using thematic analysis. The interview transcripts were systematically coded, and recurring patterns and themes were identified through an iterative analytical process reflecting participants’ perceptions and experiences of the physical work environment. Results: The analysis revealed seven main themes: impact of spatial layout on workflow; need for relaxation and break spaces; connection to nature, furniture and comfort; influence of color on mood; ambient features and environmental control; and natural light and well-being. Conclusions: This study highlights the critical role of the healthcare physical environment in shaping employee satisfaction and offers practical recommendations for healthcare facility design, emphasizing the need for ergonomic workspaces, greenspaces, and safe workplaces. This study contributes to a deeper understanding of how the physical environment can be optimized to support employees in healthcare settings.

## 1. Introduction

Staff satisfaction in healthcare facilities is a critical factor influencing the quality of care delivered to patients [1,2]. Healthcare professionals, including both clinical and nonclinical staff, often work in high-pressure environments that demand precision, resilience, and collaboration [3,4]. Within these demanding settings, the physical environment functions as an active contributor to employees’ satisfaction [5,6].

Understanding how the physical environment affects healthcare staff is increasingly important, as healthcare institutions face growing challenges related to staff burnout, retention, and operational efficiency [7,8,9]. Satisfied employees are generally more engaged, productive, and likely to remain with their organizations, contributing positively to patient care and organizational culture [10,11]. Conversely, dissatisfaction with the work environment is associated with absenteeism, higher turnover rates, and increased risk of medical errors [12,13]. Thus, improving the physical environment has the potential not only to support staff well-being but also to enhance patient safety and care quality.

Research has shown that design elements such as natural light, noise reduction, and enhanced air quality positively benefit healthcare staff [14,15,16]. These elements not only promote healing but also contribute positively to reduced staff stress and improved job satisfaction. Similarly, [17] reported that the physical environment directly impacts workplace efficiency and satisfaction among healthcare staff. Hospital layouts play a crucial role in shaping staff workflows efficiently [18,19]. Inefficient layouts that require staff to travel long distances lead to increased fatigue and reduced job satisfaction [20,21]. Conversely, a thoughtful design supporting workflow and collaboration fosters a more effective and satisfying work environment. Studies have shown that environments that include ergonomic furniture [22], efficient spatial organization [23], and access to nature [24] positively influence satisfaction. Environments with natural elements can lower stress and increase satisfaction among healthcare staff [25,26]. In line with this evidence, restorative environments such as Serenity Rooms incorporating natural light, biophilic elements, calming colors, and sensory comfort play crucial roles in promoting emotional recovery, reducing stress, and enhancing job satisfaction among nurses [27].

Perceptions of the work environment also vary by role and responsibility. Nurses, often involved in direct patient care and physically intensive tasks, may prioritize functional design [21,23] and accessibility, whereas physicians might value private and quiet spaces for communication with patients [28]. Perceived control over environmental factors such as lighting or temperature is another important determinant of staff satisfaction [29]. Addressing the unique environmental needs of staff is key to designing healthcare spaces that support satisfaction and well-being.

To investigate the relationships between staff satisfaction and their physical environment, qualitative methods using thematic analysis allow for an in-depth exploration of individual experiences. This approach is important in complex environments such as healthcare settings, where the patterns and meaning may not emerge through quantitative methods. Thematic analysis, defined by Braun and Clarke [30] as a method for identifying, analyzing, and reporting patterns within data, is valued for its flexibility and adaptability to various theoretical perspectives, making it ideal for exploring complex phenomena such as staff perceptions of the physical environment.

Despite the growing interest in workplace satisfaction within healthcare settings, much of the literature has predominantly employed quantitative methods to examine the relationship between workplace conditions and staff satisfaction and other outcomes [6,31,32]. Although qualitative approaches have been used in some studies, they have often provided limited understanding of the subjective experiences and perceptions of healthcare staff regarding the physical work environment. Consequently, the ways in which staff interpret and experience their everyday workplace settings remain insufficiently explored.

A qualitative approach, particularly thematic analysis, offers the flexibility to identify emergent themes and capture lived experiences that are frequently overlooked in survey-based research. While thematic analysis has been widely applied to understand patient experiences and to identify critical issues related to healthcare quality [33,34], its application to staff-centered investigations of the healthcare physical environment remains limited. Existing studies on healthcare staff satisfaction and workplace environments rely on quantitative designs, focus on specific professional groups, or examine isolated physical design features.

Existing studies have not integrated examinations of physical environmental dimensions such as architectural, interior, and ambient features through an explicit environment–behavior theoretical lens, particularly within low- and middle-income country contexts such as India. As a result, there is a lack of systematic understanding of how diverse healthcare staff perceive and experience the physical workplace environment and how these perceptions influence satisfaction.

To address this gap, the present study provides a theoretically grounded qualitative exploration of healthcare staff perceptions of the physical work environment, drawing on the lived experiences of both clinical and nonclinical staff. Accordingly, this study aims to explore how healthcare staff perceive and experience the physical environment of their workplace and how these perceptions shape their satisfaction.

### 1.1. Conceptual Framework

**Figure 1:**
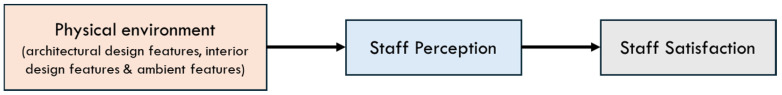
Conceptual Framework Linking the Physical Environment, Staff Perception, and Staff Satisfaction.

The conceptual framework for this study is built on the assumption that the physical environment of healthcare workplaces shapes staff perceptions, which in turn influence their overall satisfaction. The physical environment is conceptualized across three dimensions: architectural, interior and ambient features [35,36]. These environmental features form the basis of staff perceptions. Prior studies indicate that well-designed spaces can reduce stress, facilitate workflow, and enhance a sense of well-being [37,38], whereas inadequate or poorly planned settings can contribute to fatigue and dissatisfaction. Thus, the framework positions staff perceptions as the key mediating link between environmental characteristics and satisfaction, suggesting that staff are more likely to experience higher levels of satisfaction when their physical surroundings align with their work needs and promote comfort, efficiency, and psychological restoration. This conceptual structure (Figure 1) highlights the importance of staff satisfaction as a product of the dynamic interplay between environmental perceptions and user responses, suggesting that enhancing physical workplace conditions can improve morale, performance, and well-being [37,39].

### 1.2. Theoretical Framework

**Figure 2:**
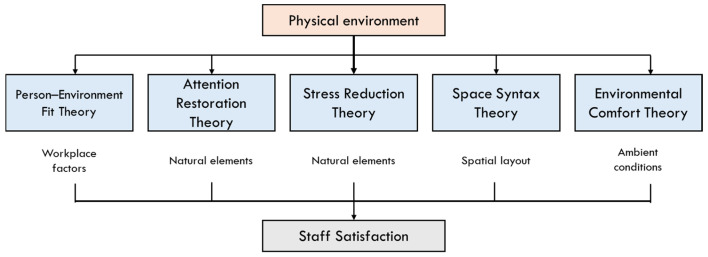
Theoretical Framework Linking Environmental Theories to Workplace Satisfaction.

The theoretical foundation of this study is informed by a set of environment–behavior and psychological theories that explain how the physical environment shapes human perception and satisfaction. Person–environment fit theory suggests that satisfaction arises when workplace environmental conditions match staff needs and expectations, explaining why ergonomic design, break spaces, and spatial layout strongly influence staff comfort and performance [40]. Attention Restoration Theory (ART) provides insight into how exposure to natural elements such as window views and greenery supports cognitive recovery and reduces mental fatigue, directly contributing to improved satisfaction [41]. This is complemented by Stress Reduction Theory (SRT), which posits that natural elements reduce physiological stress responses, aligning with staff reports that nature views and indoor plants create a calming environment [38]. Recent evidence from serenity room implementations reinforces both ART and SRT, showing that environments designed with natural elements, calming sensory input, and opportunities for psychological respite significantly enhance staff recovery and emotional regulation [27]. Space syntax theory frames how spatial configuration influences movement, workflow, and efficiency, supporting findings that poorly organized layouts hinder task performance and create congestion [42]. Environmental comfort theory further explains how ambient conditions affect users’ sense of comfort and satisfaction [39]. Together, these theories provide a strong foundation for understanding how staff perceptions of the physical environment influence their workplace satisfaction (Figure 2).

## 2. Methods

### 2.1. Study Design

This study adopted a qualitative research design to explore healthcare staff perceptions of and satisfaction with the physical environment in their workplace. The study is reported in accordance with the Consolidated Criteria for Reporting Qualitative Research (COREQ) checklist [43], which is provided in the Supplementary Material (File S1). Accordingly, thematic analysis, as outlined by [30], was used to identify and analyze patterns of meaning across participant narratives through semistructured interviews with clinical and nonclinical staff. The researchers employed a purposive sampling strategy to select participants who had relevant experience and understanding regarding the physical health care environment. This inductive approach helped generate insights grounded in the participants lived experiences rather than preconceived categories. This study was conducted by a multidisciplinary research team from a private university in India, consisting of faculty members from the Departments of Architecture, Management, and Engineering at a private higher education university in Karnataka, India. The interviews were carried out by the primary investigator, a faculty member in architecture with experience in qualitative research and a focus on how people interact with healthcare spaces. Other researchers supported the validation and the analysis part.

### 2.2. Participant Selection

The participants were selected using purposive sampling, a technique commonly employed in qualitative research to recruit individuals with direct, relevant experience of the phenomenon under investigation [44]. A purposive sampling strategy was employed to capture varied perspectives from staff representing different professional roles within the healthcare facility. The target population comprised staff from a reputed tertiary hospital in South India. The eligibility criteria included (i) current employment in the healthcare setting, (ii) a minimum of one year of experience in their present role, and (iii) willingness to participate in a face-to-face interview. The sample was intentionally composed of different professional groups (clinical and nonclinical staff) to capture diverse experiences and perceptions of the physical health care environment.

### 2.3. Ethical Considerations

The study was conducted following ethical guidelines, and ethical approval was before the commencement of the research. The participants were provided with a detailed participant information sheet explaining the purpose, procedures, and voluntary nature of the study. They were assured of confidentiality and were informed of their right to withdraw at any stage. Written consent was obtained from all participants.

### 2.4. Interview Process

Data were collected through semistructured interviews, a widely used technique in qualitative research that facilitates open-ended dialog while maintaining a focus on key areas of interest. The interview setting provided a familiar and comfortable space, encouraging participants to speak freely about their experiences. Six interview questions were designed to explore participants’ perceptions of the workplace physical environment and their satisfaction, with each main question accompanied by relevant probing questions. The semistructured interview guide and participant informed consent were finalized on the basis of feedback from two experts with experience in healthcare environment research. Data were collected using semi-structured interviews guided by a predefined interview guide (Supplementary Material File S2). This approach allowed for a flexible, conversational style of engagement, offering space for participants to raise issues not directly addressed in the prepared questions but relevant to their lived experience. The interview questions were theoretically informed by established frameworks in environmental psychology and spatial analysis that explain the relationship between the physical environment and the user experience. Specifically, PE-fit theory guided questions examining staff perceptions of compatibility between workplace design features and functional needs, as well as overall satisfaction with the physical environment [40]. ART and SRT provide answers to questions exploring emotional responses, happiness, comfort, and perceived restorative or stress-inducing qualities of architectural, interior, and ambient features within the workplace [45]. In addition, Space Syntax Theory underpinned questions related to spatial layout, circulation, visibility, and accessibility, capturing how spatial configuration influences everyday work experiences and interactions [42]. Together, these theories provided a robust conceptual basis for the interview guide, ensuring that the questions systematically captured the cognitive, affective, and experiential dimensions of healthcare staff–environment interactions.

### 2.5. Data Analysis

The interview recordings were transcribed, and the data were analyzed using thematic analysis in accordance with [28]. The data from the transcripts were coded by one primary coder, with the support of two secondary coders, to identify initial codes. Any disagreements in coding were resolved through discussion until consensus was reached. Reflexive memos were maintained throughout the analysis to capture analytic decisions and emerging insights, and an audit trail was also held to record the coding process, theme development, and any modifications made during analysis, thereby enhancing transparency and rigor [46]. The transcripts were read repeatedly to achieve familiarization with the data, after which manual coding was performed to identify recurrent patterns. Related codes were grouped into subthemes representing significant meanings, which were subsequently organized into overarching themes. The analysis focused on how staff perceive, experience, and emotionally respond to the physical features of their work environment. This approach enabled the identification of key design aspects that were perceived as either supportive of or detrimental to staff well-being, contributing to a holistic understanding of staff–environment interactions in healthcare settings.

## 3. Results

The study included ten participants, comprising six clinical staff—two doctors (MDs), three nurses (Ns) and one lab assistant (LA)—and four nonclinical staff members—two administrative staff (ASs), one receptionist (R) and one pharmacist (P). All participants had a minimum of two years of experience in the hospital. The gender ratio of the participants was 4:6 (male to female), with ages ranging between 25 and 48 years.

Data saturation was assessed iteratively during data collection and analysis. After the ninth interview, no new codes or themes emerged, and the tenth interview confirmed thematic redundancy. Saturation was therefore deemed achieved when successive interviews failed to generate new themes [47,48].

**Figure 3:**
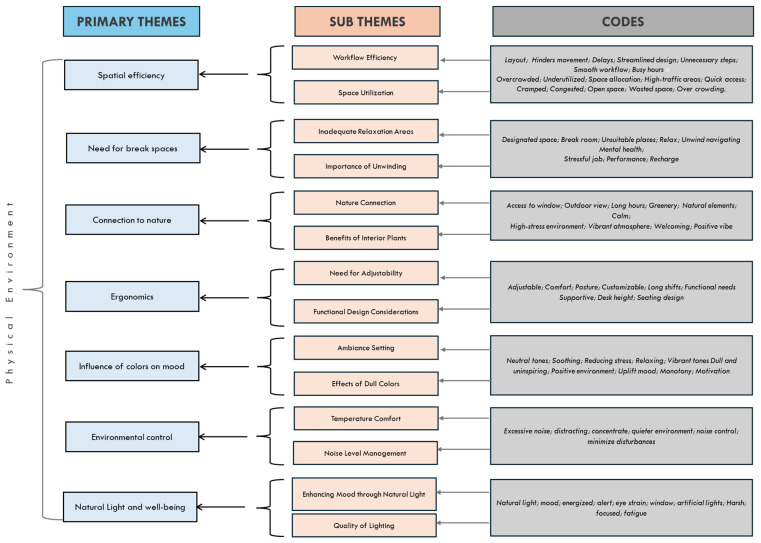
Diagrammatic representation of the major themes, subthemes and codes.

Thematic analysis revealed seven primary themes related to their perceptions of the physical environment. To account for differences in professional roles, patterns were examined both within and across clinical and nonclinical staff. Codes and themes were compared across professional groups to identify perceptions, thereby minimizing the influence of professional role as a confounding factor. The themes presented reflect consistent patterns across both clinical and nonclinical staff, supported by representative participant quotations. Figure 3 presents the primary themes and corresponding subthemes and codes, with subthemes illustrated with participant quotations.

### 3.1. Theme 1: Spatial Efficiency

Spatial efficiency has emerged as a central factor influencing workflow effectiveness, particularly for clinical staff whose roles require rapid movement, spatial coordination, and uninterrupted task execution. While both clinical and nonclinical participants recognized the importance of an organized spatial layout, their interpretations differed markedly. Clinical staff consistently linked spatial inefficiencies to disruptions in patient care processes, whereas nonclinical staff primarily perceived these issues as sources of inconvenience and operational discomfort. This difference illustrates how professional roles shape the perceived significance of spatial design limitations.

#### 3.1.1. Subtheme 1.1: Workflow Efficiency

Clinical staff frequently identified congestion, poorly planned circulation routes, and inadequate proximity between functional zones as major impediments to efficient care delivery. These spatial constraints were not viewed merely as design shortcomings but as factors contributing to care delays, increased cognitive burden, and heightened stress, particularly during peak operational periods. In contrast, although nonclinical staff acknowledged movement-related challenges, they did not associate these constraints strongly with time-critical outcomes or patient safety.

*“The current layout hinders our movement between patient stations, leading to delays in providing care. We need a more efficient design to reduce unnecessary steps…. feels like we’re wasting time navigating the workspace than focusing on patient needs.”*

(N 1)

#### 3.1.2. Subtheme 1.2: Space Utilization

Proper space utilization is essential for maintaining an effective workflow. Ineffective space utilization further exacerbates workflow challenges. The participants from both groups reported overlapping functional areas and the absence of clearly designated zones; however, the implications attributed to these conditions differed. Clinical staff emphasized the impact on patient interaction, privacy, and task concentration, whereas nonclinical staff framed these issues largely in terms of organizational confusion and reduced operational clarity.

*“Some areas are overcrowded while some are underutilized…. the space allocation can be better designed, need to utilize space better, especially in frequently used areas such as supplies”*

(LA 1)

Consistent differences emerged between clinical and nonclinical staff perceptions of the physical environment. Clinical staff prioritized spatial efficiency, accessibility, and environmental control because of their direct impact on patient care and task performance, whereas nonclinical staff placed greater emphasis on comfort, esthetics, and ambient conditions. These contrasts reveal how professional roles mediate environmental experience and highlight the importance of role-sensitive design strategies in healthcare settings.

### 3.2. Theme 2: Need for Break Spaces

Relaxation spaces are critical for staff well-being, providing places for rest and psychological recharge. The participants emphasized that access to private and quiet break spaces is essential for maintaining emotional balance and preventing work-related fatigue.

#### 3.2.1. Subtheme 2.1: Inadequate Relaxation Areas

The respondents expressed dissatisfaction with the lack of designated relaxation spaces within the hospital. In the absence of formal break areas, staff reported resorting to makeshift areas such as storerooms, corner spaces, and unused corridors for breaks. This spatial inadequacy constrained opportunities for meaningful rest and was perceived to negatively affect their ability to recover during work hours, contributing to emotional exhaustion and reduced job satisfaction.

*“There’s no space for a quick break. We end up resting in unsuitable places such as corridors or corner spaces. When we take short breaks, we find it difficult to find a comfortable space to relax…”*

(N 2)

#### 3.2.2. Subtheme 2.2: Importance of Unwinding

The findings further highlight the importance of dedicated spaces that enable staff to mentally disengage from continuous clinical demands. The participants indicated that even modest, purpose-designed relaxation areas could facilitate brief periods of unwinding, allowing them to return to work with improved focus and resilience. The lack of such spaces was perceived as limiting the restorative value of short breaks, particularly in high-intensity work settings.

*“After attending patients back-to-back, a proper space to unwind becomes essential. Taking short breaks between hectic schedules is important, but without a peaceful space, I don’t feel refreshed.”*

(MD 1)

The findings indicate a pronounced need for dedicated break and relaxation spaces within the hospital workplace. The absence of adequately designed and accessible relaxation areas compelled staff to rely on improvised spaces, limiting opportunities for effective rest and recovery. This inadequacy was perceived to adversely influence staff well-being, contributing to fatigue, emotional strain, and diminished work satisfaction. The participants consistently emphasized that even small, purpose-designed break spaces could play a significant restorative role, support mental rejuvenation and sustain work performance in demanding healthcare environments.

### 3.3. Theme 3: Connection to Nature

Connection to nature emerged as an important environmental attribute influencing staff well-being, particularly in mitigating stress within demanding healthcare settings. The participants described access to natural elements, such as outdoor views and interior plants, as contributing to a more calming and supportive work environment. While both clinical and nonclinical staff valued exposure to nature, clinical staff more frequently emphasized its role in emotional regulation and stress relief during prolonged and high-pressure work periods.

#### 3.3.1. Subtheme 3.1: Nature Connection

Access to outdoor views, particularly views of greenery, was perceived as a source of psychological relief. Staff reported that visual connection to the outdoors helped alleviate mental fatigue and provided brief moments of restoration during long working hours. This finding suggests that window views serve not only as esthetic features but also as restorative elements that support emotional well-being in high-stress healthcare environments.

*“Having access to a window with an outdoor view would truly help during long hours of work… Seeing greenery around brings a sense of calmness, especially in a high-stress environment.”*

(AS 1)

#### 3.3.2. Subtheme 3.2: Psychological Benefits of Interior Plants

Interior plants were described as enhancing overall workplace ambiance by reducing the clinical feeling of the environment and fostering a more welcoming atmosphere. The participants associated the presence of greenery with improved comfort, perceived air quality, and emotional ease. These perceptions indicate that interior planting contributes to psychological well-being by introducing biophilic elements that soften the institutional character of healthcare spaces.

*“Plants brighten the room, making it feel more alive and less clinical. I feel more at ease working in a space with greenery; it creates a positive vibe.”*

(MD 2)

The findings indicate that connection to nature is a significant environmental factor influencing healthcare staff well-being. Access to outdoor views and the presence of interior plants were consistently perceived as restorative elements that alleviate stress, reduce mental fatigue, and enhance emotional comfort in demanding work settings. These natural features contributed to creating a supportive workplace atmosphere, with particular relevance for staff working long hours in high-pressure environments. The results highlight the importance of integrating biophilic elements within healthcare facilities to support staff’s psychological well-being and improve the quality of the work environment.

### 3.4. Theme 4: Ergonomics

Ergonomics emerged as a significant factor influencing staff comfort, physical well-being, and work efficiency. Furniture design was not merely perceived as a matter of comfort but also as a determinant of sustained work performance and physical strain. Differences in emphasis were observed between professional roles and nonclinical staff, particularly administrative personnel, who expressed greater sensitivity to prolonged sedentary work and its ergonomic implications.

#### 3.4.1. Subtheme 4.1: Need for Adjustability

The need for adjustable furniture was strongly articulated, especially by administrative staff, who spend extended periods at workstations. The participants described existing furniture as lacking basic ergonomic features, such as adjustable seating height and desk flexibility, leading to discomfort, poor posture, and musculoskeletal strain. These accounts suggest a misalignment between furniture design and the physical demands of routine work tasks, highlighting a person–environment mismatch that may contribute to fatigue and reduced productivity.

*“The chairs and desks aren’t adjustable, which makes it uncomfortable during long shifts. The chair height can’t be adjusted, making it hard to maintain good posture during work… It would help if the desks were adjustable to fit different tasks, whether we’re sitting or standing.”*

(AS 2)

#### 3.4.2. Subtheme 4.2: Functional Design Considerations

In addition to adjusting, the participants emphasized the importance of furniture that supports multiple work functions. Staff expressed dissatisfaction with furniture that prioritized appearance over usability, noting that current designs often fail to provide adequate support for long-duration tasks. This concern was particularly evident among nonclinical staff engaged in desk-based work, where insufficient ergonomic support was perceived to exacerbate physical discomfort and reduce work efficiency.

*“We spend hours at the desk, but the seating isn’t designed for long-term comfort, and the current furniture design just isn’t supportive enough.”*

(P 1)

These findings highlight the need for ergonomically and functionally responsive furniture that accommodates diverse task requirements and prolonged use, reinforcing the role of ergonomic design in promoting staff well-being and operational efficiency.

### 3.5. Theme 5: Influence of Colors on Mood

Color emerged as a significant design feature shaping staff moods and emotional comfort within the workplace environment. The participants consistently associated color schemes with their psychological states, noting that softer and cooler tones were perceived as calming and stress-reducing, whereas brighter colors were viewed as energizing. These perceptions suggest that color selection contributes not only to esthetic appeal but also to staff well-being and sustained focus during work activities.

#### 3.5.1. Subtheme 5.1: Ambiance Setting

Staff highlighted the role of color in establishing the overall ambience of the workplace. Softer and balanced color palettes were perceived to create a calming atmosphere that supported concentration, particularly during high-pressure tasks. Clinical staff associated calming color schemes with improved focus and emotional regulation during demanding procedures.

*“Soft colors make the environment more relaxing and calming, which helps focus during procedures… which is helpful in reducing stress during high-pressure moments.”*

(N 3)

#### 3.5.2. Subtheme 5.2: Effects of Dull Color

Some staff noted that dull or overly neutral color palettes contribute to feelings of boredom and fatigue, while incorporating vibrant colors helps create a more dynamic and stimulating environment and could increase motivation and engagement levels. The participants suggested that the strategic use of more vibrant or varied colors could enhance stimulation, uplift mood, and counteract monotony in routine work settings.

*“The lack of color variation makes the workspace feel dull and uninspiring… the workspace colors feel bland… A more active color could help uplift the mood and break the monotony.”*

(N 4)

The findings indicate that workplace color schemes play a significant role in shaping healthcare staff’s emotional responses, concentration, and perceived comfort. Calming colors were associated with reduced stress and enhanced focus, particularly during high-pressure clinical tasks, whereas monotonous or dull color palettes were linked to mental fatigue and reduced motivation. The results highlight a clear need for balanced color strategies that support emotional regulation without compromising visual stimulation, underscoring color as a functional design element influencing staff well-being and work engagement in healthcare environments.

### 3.6. Theme 6: Ambient Features and Environmental Control

Ambient environmental features emerged as critical determinants of staff comfort and overall workplace satisfaction. The participants consistently highlighted temperature regulation and noise control as key factors influencing their ability to concentrate and perform tasks effectively. Well-regulated ambient conditions were perceived as supportive of both physical comfort and psychological well-being, whereas poorly controlled conditions were associated with distraction, fatigue, and increased stress.

#### 3.6.1. Subtheme 6.1: Temperature Comfort

The perceived comfort of workplace temperature was strongly linked to staff satisfaction and task engagement. The participants reported that limited control over temperature settings and frequent fluctuations disrupted their comfort and focus during work hours. These conditions were described as particularly challenging during prolonged work periods, contributing to physical discomfort and reduced concentration.

*“The temperature fluctuates a lot, and it’s hard to maintain a consistent comfort level throughout the day… temperature control could be better and sometimes it gets too cold, affecting our comfort.”*

(AS 3)

#### 3.6.2. Subtheme 6.2: Noise-Level Management

Noise was identified as a prominent environmental stressor, particularly in relation to equipment sounds, conversations, and interactions involving patients and their families. The participants noted that excessive noise levels interfered with concentration and heightened stress, especially in work areas requiring sustained attention. The need for design strategies that mitigate noise disturbances was emphasized as essential for improving focus and maintaining a supportive work environment.

*“The noise from the equipment can be distracting, especially when trying to concentrate… noise from patients and families makes it hard to concentrate and focus. A quieter environment would help improve focus.”*

(LA 2)

Overall, ambient features such as temperature regulation and noise control play pivotal roles in shaping staff comfort, concentration, and workplace satisfaction. Staff experience highlighted that inadequate control over these environmental factors acts as a source of distraction and stress, particularly in high-demand clinical areas. Conversely, well-managed ambient conditions were associated with improved focus, reduced stress, and increased overall satisfaction. These findings highlight the importance of integrating effective environmental control strategies into healthcare workplace design to support both physical comfort and cognitive functioning.

### 3.7. Theme 7: Natural Light and Well-Being

Natural light has emerged as a key factor influencing healthcare staff well-being. The participants consistently highlighted that abundant natural light positively affected focus, alertness, and overall comfort, contributing to both mental and physical health in high-stress healthcare settings. Clinical staff, whose work demands sustained attention and vigilance, particularly emphasized the restorative and energizing effects of daylight exposure, whereas nonclinical staff framed its importance in terms of mood and general comfort. These findings suggest that access to natural light is a critical environmental feature that supports both functional performance and emotional well-being.

#### 3.7.1. Subtheme 7.1: Enhancing Mood Through Natural Light

Natural light is regarded as a vital component of a healthy work environment. Staff reported that exposure to natural light improves mood and energy levels and enhances overall well-being. The presence of large windows was mentioned as a critical factor that positively impacts their daily experiences at work. This highlights the dual benefit of natural light: promoting psychological restoration while supporting sustained cognitive performance, particularly for staff engaged in high-intensity tasks.

*“Natural light truly helps during long shifts when I’m feeling tired… having access to sunlight makes a huge difference in how energized I feel during the day. it reduces eye strain also.”*

(MD 3)

#### 3.7.2. Subtheme 7.2: Lighting Quality

Staff also noted that artificial lighting quality affects comfort and visual performance. Harsh or insufficient lighting has been reported to cause eye strain and discomfort, emphasizing the need for adjustable and well-designed artificial lighting to complement natural daylight.

*“They can think of using better lighting in some areas. The artificial lights are too harsh, especially during long hours.”*

(Receptionist)

Overall, both natural and high-quality artificial lighting were perceived as critical contributors to staff well-being, influencing mood, energy, focus, and visual comfort. Access to daylight and well-designed lighting solutions not only enhances daily work experiences but also supports mental and physical health, particularly for clinical staff engaged in high-intensity tasks. These findings highlight the importance of lighting as a key environmental factor in healthcare workplace design.

## 4. Discussion

The thematic analysis revealed seven major themes and associated subthemes. Each theme presented an important understanding of how the aspects of the physical environment impacted staff. The spatial layout directly affects participants’ ability to function efficiently, highlighting its importance. The subtheme workflow efficiency highlighted that inefficient layouts disrupted smooth transitions between workstations and increased the risk of errors. This finding supports earlier findings that spatial design reduces operational delays in healthcare environments [20,21,49]. Under the subtheme space utilization, staff reported that overlapping or underutilized zones caused congestion or confusion. These observations are consistent with space syntax theory, which relates user behavior and efficiency to spatial configuration [50]. Efficient space planning could significantly improve healthcare delivery outcomes and reduce stress levels among staff [51]. In addition to providing benefits to staff well-being and satisfaction, supportive physical work environments may also play an important role in enhancing the quality and safety of patient care.

The staff strongly emphasized that dedicated relaxation areas were essential for workplace satisfaction. Dissatisfaction with the absence of relaxation areas is reflected in the subtheme of inadequate relaxation areas. These findings are consistent with previous research suggesting that poorly designed break spaces lead to burnout and lower job satisfaction [52,53]. The findings concerning the importance of unwinding suggest that access to quiet, comfortable spaces can increase morale, cognitive recovery, and performance. These findings align with recent syntheses showing that restorative environments, specifically serenity rooms designed for nurses, improve emotional balance, reduce stress, and facilitate psychological recovery through biophilic design, calming colors, and sensory modulation [27], a relationship further supported by ART, which proposes that such environments support recovery from mental fatigue [54].

The participants highlighted the importance of biophilic design elements. Staff frequently mentioned the psychological benefits of having visual access to natural elements, as captured in the subtheme nature connection. This confirms earlier findings that views of nature reduce perceived stress and enhance emotional resilience among healthcare workers [45]. The subtheme benefits of interior plants also highlight the impact of greenery on mood and ambiance. Stress reduction theory [38] explains how natural elements can reduce physiological stress responses and improve psychological functioning in healthcare environments.

Ergonomics has emerged as crucial to staff health and efficiency. Under the subtheme need for adjustability, staff pointed out how the lack of ergonomic furniture contributed to physical discomfort and long-term strain. These findings are consistent with studies that stress the importance of customized workstations in preventing musculoskeletal disorders and enhancing productivity [55,56]. With respect to functional design considerations, staff favor furniture that supports both functional requirements and esthetic standards. PE fit theory suggests that using adjustable, task-appropriate furniture improves the match between a person’s physical ability and their environment, leading to better comfort and satisfaction.

This study demonstrated the psychological impact of color on mood and energy levels. As observed in the subtheme ambiance setting, calming color schemes were preferred for promoting tranquility and focus. These findings align with color psychology research, which indicates that certain hues can have soothing effects, thereby enhancing performance and reducing anxiety [57]. The effects of dull colors showed that color schemes that are neutral can make staff feel less motivated. Within environmental comfort theory, color contributes to psychological comfort, shaping affective appraisals and perceived environmental support. When color palettes align with the task context and emotional demands, PE fit is strengthened, supporting satisfaction.

Ambient environmental features such as temperature, noise, and lighting significantly influence staff satisfaction. Under temperature comfort, staff explained how inadequate temperature regulation interferes with their comfort, which is supported by studies indicating that thermal comfort is directly linked to workplace satisfaction [32,58]. Concerns regarding distracting noise from equipment, conversations, and patient activities were emphasized in subtheme noise level management. High noise levels have long been associated with increased stress and fatigue in healthcare settings [59,60,61]. Environmental comfort theory offers a comprehensive account of these effects by specifying how ambient conditions shape perceived comfort, cognitive load, and satisfaction. Moreover, PE fit suggests that when ambient parameters align with staff preferences and task requirements, perceived fit and well-being improve.

The findings concerning natural light and well-being reaffirm the proven relationship between daylight exposure and improved satisfaction [14,62,63]. Daylight aligns with ART and SRT through stress mitigation through positive affective cues, providing both cognitive recovery and affective benefits [38,41]. It also contributes to environmental comfort through circadian support and visual quality, enhancing PE fit when illumination levels match task needs.

While the findings align with research from high-income settings, they have particular relevance for low- and middle-income healthcare facilities, such as those in India. Resource constraints, high patient loads, and infrastructural limitations can exacerbate the impact of suboptimal spatial layouts, inadequate relaxation spaces, and poor lighting on staff well-being and workflow efficiency. These results highlight the importance of context-sensitive, cost-effective design strategies that increase staff satisfaction and performance in resource-limited healthcare settings.

## 5. Conclusions

The study explored healthcare staff perceptions of their physical work environment and identified seven themes: spatial layout, relaxation and break spaces, connection to nature, furniture comfort, color and mood, ambient features, and natural light. Together, these themes offer a comprehensive account of how the physical environment influences staff satisfaction and well-being in hospital settings. For example, inefficient spatial layouts are linked to workflow disruptions, whereas well-zoned spaces enhance operational efficiency, findings that are consistent with space syntax theory on the role of spatial configuration in movement and task performance. The lack of adequate relaxation areas contributed to emotional fatigue, highlighting the need for quiet, restorative spaces that support recovery, as explained by ART and SRT. The presence of natural elements and outdoor views was associated with reduced stress and improved mood, reflecting the biophilic and restorative design principles grounded in ART and SRT. Ergonomically designed furniture has emerged as a crucial component of user-centered design; in PE fit terms, adjustable and task-appropriate furnishings enhance the fit between environmental demands and staff capabilities. Color schemes shaped emotional states and focus, contributing to psychological comfort within the Environmental Comfort Theory. Ambient features were directly linked to comfort, concentration, and satisfaction, aligning with Environmental Comfort Theory and reinforcing PE Fit when conditions meet staff needs.

Collectively, the results indicate that the physical environment does not merely function as a backdrop; it actively shapes experiences, performance, and well-being through theoretically grounded mechanisms: fit (PE Fit), restoration (ART), stress mitigation (SRT), spatial affordances (Space Syntax), and comfort (Environmental Comfort). For designers and administrators, this implies prioritizing staff needs alongside patient needs by optimizing layout efficiency (Space Syntax), integrating biophilic features and daylight (ART/SRT), improving furniture ergonomics (PE Fit), curating calming color palettes (Environmental Comfort), and managing ambient conditions such as temperature, noise, and lighting (PE Fit). Engaging staff in participatory design ensures that improvements align with their actual needs, strengthening perceived fit and, in turn, satisfaction and resilience.

The practical implications are as follows: optimize spatial configuration to reduce congestion and errors; provide dedicated, restorative break spaces; include biophilic elements and direct access to daylight; deploy adjustable, ergonomic furniture; curate color schemes that support the emotional tone of work areas; and control ambient conditions to enhance comfort and cognitive performance. These steps, grounded in the five theories, can improve satisfaction. Grounded in the theoretical frameworks underpinning this study, these strategies have the potential to improve staff satisfaction and well-being in healthcare settings.

This study has some limitations. First, the use of face-to-face interviews may have introduced social desirability bias, as participants could have moderated their responses when discussing workplace conditions, potentially leading to more favorable or restrained accounts. Second, although comparisons were examined across clinical and nonclinical staff, the small subgroup sizes limited a more detailed exploration of role-specific differences, which may have obscured nuanced variations in perceptions across professional groups. Third, the study relied solely on self-reported data, without triangulation through observational methods or objective environmental measures, which may have influenced the depth and robustness of interpretations. Finally, the data were collected from a single hospital, limiting the generalizability of the findings. Future research should include multiple healthcare facilities, larger and more balanced professional subgroups, and methodological triangulation using observational, spatial, or environmental performance measures. Mixed-method, longitudinal, or intervention-based designs would further strengthen causal inference and enhance a reflective understanding of how physical environment features influence staff well-being and performance.

## Data Availability

Due to privacy and confidentiality concerns related to the interview content, the interview transcripts cannot be made publicly available, while representative interview excerpts have been included in the manuscript, and any reasonable inquiries regarding the interview data may be directed to the corresponding author.

## Supplementary Materials

The following supporting information can be downloaded at: https://www.mdpi.com/article/10.3390/healthcare14050642/s1, File S1: COREQ checklist; File S2: Semistructure interview guide, Participant Information Sheet & Informed Consent
