# Supplementary material for: Exploring Healthcare Staff Perceptions and Satisfaction with the Physical Work Environment: A Qualitative Study
Source: Healthcare (Basel). 2026 Mar 4;14(5):642. doi: 10.3390/healthcare14050642 (PMC12984282; doi:10.3390/healthcare14050642)
Supplement: Supplementary file 1 [file healthcare-14-00642-s001.zip › healthcare-4062504-supplementary.pdf]

Supplementary FileA:

Consolidated criteria for reporting qualitative studies (COREQ): 32-item checklist

No Item		Guide questions/description
Domain 1: Research team and reflexivity		
Personal Characteristics		
1	Interviewer/facilitator	Interviews were conducted by the primary researcher.
2	Credentials	The corresponding authors are PhD qualified academicians with research experience in healthcare environment and workplace studies.
3	Occupation	The researchers were academic researchers at the time of the study.
4	Gender	The researcher was female.
5	Experience and training	The researchers are experienced in qualitative research and interview-based studies.
Relationship with participants		
6	Relationship established	No prior relationship with participants.
7	Participant knowledge of the interviewer	Participants were informed about the purpose of the research.
8	Interviewer characteristics	Demonstrated interest and research experience in healthcare workplace environments.
Domain 2: study design		
Theoretical framework		
9	Methodological orientation and Theory	Qualitative descriptive approach using semi-structured interviews.
Participant selection		
10	Sampling	Purposive sampling
11	Method of approach	Participants were approached in person.
12	Sample size	Ten participants
13	Non-participation	None
Setting		
14	Setting of data collection	Hospital
15	Presence of non-participants	None
16	Description of sample	clinical staff (two doctors, three nurses and one lab assistant) and nonclinical staff (two administrative staff, one receptionist and one pharmacist).
Data collection		
17	Interview guide	Semi-structured interview guide used.
18	Repeat interviews	No
19	Audio/visual recording	Audio not recorded.
20	Field notes	Notes were taken during the interview.
21	Duration	Approximately 30–45 minutes
22	Data saturation	Data saturation was discussed and confirmed through consensus among the corresponding author and co-authors.
23	Transcripts returned	Transcripts were not returned to participants.
Domain 3: analysis and findings		
Data analysis		
24	Number of data coders	Data were coded by one primary coder with support from two secondary coders.

25	Description of the coding tree	The coding tree was developed inductively, with codes and themes emerging from repeated patterns identified in the interview data.
26	Derivation of themes	Themes were derived using a data-driven approach, based on patterns identified in the interview transcripts.
27	Software	Manual thematic analysis.
28	Participant checking	Not conducted
Reporting		
29	Quotations presented	Yes, representative quotations included.
30	Data and findings consistent	Yes
31	Clarity of major themes	Clearly presented
32	Clarity of minor themes	Minor themes are clearly presented and supported by representative participant quotations.

Supplementary FileB: Semi-Structured Interview Guide

The following interview guide is used for qualitative data collection in private hospitals.

Participant Code	
Interview Date (dd/mm/yyyy)	
Audio Recording Consent	□ Yes □ No
Interview Start Time	
Interview End Time	

To read to the participants at the beginning of the interview.

Thank you for agreeing to participate in this interview. This study explores healthcare staff perceptions of the physical work environment and its influence on staff satisfaction in private hospitals.

Participation in this interview is entirely voluntary. You may choose not to answer any question or withdraw from the interview at any time without any consequences.

With your permission, the interview will be audio-recorded. If you prefer not to be recorded, notes will be taken instead. The interview is expected to last approximately 25 minutes.

All information shared during this interview will be kept confidential and used solely for research purposes. No identifying information will be included in any reports or publications.

Background Information

- Professional role/designation: __________
- Department/unit: _________
- Years of experience in this hospital: ___________
-

Interview Questions

1. Can you describe the physical environment of your workplace?
   Probe: What physical features surround your work area?
2. Which aspects of your workplace environment contribute positively to your mood or well-being?
   Probe: Why do these features make you feel positive? How do you interact with them during work?
3. Are there any design elements in your workplace that you would like to change, improve, or better control?
   Probe: Please consider architectural, interior, and ambient features.

4. Overall, how satisfied are you with your workplace physical environment?
   Probe: Which aspects contribute most to your satisfaction?
5. What do you like most and least about your workplace physical environment?
   Probe: Why are these features important to you?
6. If you were given the opportunity, what changes would you propose to improve the workplace environment?
   Probe: Please elaborate on how these changes could support your work or well-being.

Appendix A: Participant Information Sheet (PIS)

Title: Assessment of Employee Satisfaction with the Physical Environment in Private Hospitals

Please read this information carefully. If you decide to volunteer to take part in this study.

We invite you to take part in our research study. Before you decide whether to participate, you should understand why the research is being done and what it will involve. Please take your time to read the following information carefully and feel free to ask if you need more information or if there is anything that you do not understand. Please also feel free to discuss this with your friends, relatives and anyone else you wish.

What is the purpose of the study?
The study aims to explore how the physical hospital environment influences staff satisfaction. As a part of the qualitative study, a semi-structured interview is used as a method of data collection that involves an interviewer and the interviewee discussing about the research topic. The purpose of the research is to gain insight into certain aspects of the workplace physical environment from the employees working in private hospitals using a semi-structured interview.

Why have I been chosen to take part?
You have been invited to participate in this study because you have at least two years of work experience at this hospital.

Do I have to take part?
Participation in this study is entirely voluntary. You are free to decide whether or not to take part. If you choose to participate, you will be asked to sign an informed consent form. If you initially agree to participate but later change your mind, you are free to withdraw from the study at any time without providing a reason.

What will happen if I take part?
If you agree to participate, you will take part in an interview to share your experiences and perceptions of the hospital's physical work environment.

Where will the interview take place?
The participants will be interviewed individually in their respective workplace in the hospital.

Are there any risks in taking part?
We do not expect any risks or discomfort associated with this research study. However, if you feel uncomfortable, you can stop the interview at any time, without giving a reason.

Will my participation be kept confidential?

All the information you give us will be kept confidential. Only the researcher will have access to what you have said. The audio recording of the interview will be identified with a code number. The audio recording will be transcribed, and identifying details (name of the interviewee, location and other details) will be removed from the transcripts.

What will happen to the information?
Following completion of the study, anonymized data from the interviews may be used in a journal publication. No names or identifying details will be included in any reports or publications. Access to the full dataset will be restricted to the researcher only.

What will happen if I want to stop taking part?
If you decide to withdraw from the study at any stage, you may do so without giving a reason. You may also request that your data be removed from the study and destroyed.

What happens next?
If you would like to take part in the study, please read and complete the consent form. Thank you for considering participation in this research.

Appendix B: Informed Consent Form

I understand that my participation in this interview is voluntary, and I can decline participation without giving any reason. I have been given sufficient information to review the information and have sought the required clarification. I understand that I am allowed to take a screenshot of the form for my reference.

By ticking the "☐" button, I give my consent for the study.

- ☐ I confirm that I have read and understood the Participant Information Sheet.
- ☐ I understand participation is voluntary and that I am free to withdraw from the project at any time, without having to give a reason and without any consequences.
- ☐ I understand anonymised data may be used in publications.
- ☐ I consent to participate in this research study.
- ☐ I consent to the interview being audio recorded as part of the research study.

Participant Name: ______________________________

Signature: ____________________________________

Date: ____
